# Scaling Up a Strengthened Youth-Friendly Service Delivery Model to Include Long-Acting Reversible Contraceptives in Ethiopia: A Mixed Methods Retrospective Assessment

**DOI:** 10.15171/ijhpm.2019.76

**Published:** 2019-09-28

**Authors:** Fariyal F. Fikree, Habtamu Zerihun

**Affiliations:** ^1^Evidence to Action/PATH, Washington, DC, USA.; ^2^Pathfinder International, Addis Ababa, Ethiopia.

**Keywords:** Family Planning, Youth-Friendly Services, Scaling-up, Mixed Methods Study, Ethiopia

## Abstract

**Background:** Donor funded projects are small scale and time limited, with gains that soon dissipate when donor funds end. This paper presents findings that sought to understand successes, challenges and barriers that influence the scaling up and sustainability of a tested, strengthened youth-friendly service (YFS) delivery model providing an expanded contraceptive method choice in one location – the YFS unit – with additional units in Amhara and Tigray, Ethiopia.

**Methods:** This retrospective mixed methods study included interviews with key informants (KIs) (qualitative arm) and analysis of family planning (FP) uptake statistics extracted from the sampled health facilities (quantitative arm). A multistage convenience purposive sampling technique was adopted to randomly select 8 health facilities aligned with respective woredas, zones and regional health bureaus (RHBs). A semi-structured interview guide soliciting information on 6 scaling-up elements (stakeholder engagement, roles and responsibility, policy environment, financial resources, quality of voluntary FP services and data availability and use) guided the interviews. Fifty-six KI interviews were conducted with policy-makers, program managers, and clinic staff. Recurring themes were triangulated across administrative levels and implementing partners. Relevant FP data (acceptor status, age and method uptake) were extracted from the 8 sampled health facilities for a thirteen-month period. Qualitative findings triangulated with FP service statistics assessed the influence of the 6 scaling-up elements with trends in long-acting reversible contraceptive (LARC) uptake before and after training.

**Results:** Our findings depict that respondents were knowledgeable and supportive of an expanded method mix. Statistically significant increases in long-acting contraceptive uptake were noted at 2 of the 8 health centers. Fidelity to the tested model was operationally constrained; respondents frequently mentioned trained staff absences and turnover as obstacles in offering quality FP services.

**Conclusion:** Despite conducive policy environment, supportive stakeholders, favorable environment, and financial support for trainings, statistically significant increases in LARC uptake occurred at only 2 of the 8 health centers; indicating the influence of weak health systems, poor quality of voluntary FP services and a ceiling effect. Scale-up processes must consider potential bottlenecks of weak health systems and availability of financial resources by addressing these as crucial elements in any systematic scale-up framework.

## Background

There are a wealth of evidence-based tools and approaches for strengthening reproductive health (RH) services. However, a significant gap exists between the evidence available and the degree to which the evidence is used to implement RH services at scale. The inability to scale up evidence-based interventions impeded several low- and middle-income countries (LMICs) from achieving the Millennium Development Goals, and many LMICs continue to make limited progress in attaining health-related Sustainable Development Goals.

Projects led by non-governmental organizations (NGOs) and funded by international donors are small scale and time limited, with transitory gains that soon dissipate when the project concludes and/or donor funds dry up. Despite limited success in implementing RH programs at scale, growing attention to scaling up best practices has resulted in analytic frameworks, strategic planning, and implementation tools aimed at facilitating scale-up.^1-4^ These resources are intended to be used to scale up small-scale projects that have proven positive outcomes nationally. Achieving universal health coverage and Sustainable Development Goal 3 is only possible through national scale-up of these proven health and RH programs.

Lack of political commitment, strong leadership, and service delivery capacity (technical and financial) hinder successful scale-up.^1-5^ Studies show that sustained commitment, government ownership, good governance, leadership, financial investments, well-coordinated donor support, and strengthened health systems (particularly human resources and commodity security) are key factors influencing effective national scale-up.^4-15^ For example, in Ethiopia, Kyrgyzstan, and Bangladesh, strong political commitment aligned with good governance, coordinated donor support, and the ability to adapt to resource limitations and competing priorities within constrained and weak health systems have contributed to improving health outcomes.^6,12^ In Nigeria, there was near nation-wide scale-up of a school-based comprehensive sexuality education program supported by strong political leadership, although lack of a predictable funding source and competing priorities for available human resources were recognized as major impediments for sustainability.^5^ In Bolivia, institutionalizing and scaling up post-abortion care was achieved by improving health system capacity through training, supervision, development of post-abortion care guidelines, and access to essential technologies.^13^

To meet global family planning (FP) goals, RH programs, particularly for youth, need to be scaled up. At the 2012 FP2020 Summit, a global agenda was set for expanding access to FP information, services, and supplies to an additional 120 million women and girls. The mid-term review in 2017 reinforced the global agenda with dedicated FP investments and programming to address high unmet need among youth.^16^ Poor RH outcomes among young people under 25 are indicative of numerous barriers that they face in accessing RH services, including contraception.

In 2016, 23 million adolescents had an unmet need for modern contraception and were at a high risk of unintended pregnancy.^17^ In 42 sub-Saharan Africa countries, close to half of the estimated 42 million unintended births were to youth.^18^ To meet the growing RH needs of over 1.2 billion young people globally and to reduce unintended pregnancy, unsafe abortions, and maternal mortality it is essential to scale-up youth-friendly services (YFSs) including quality contraceptive services. A few LMICs—Moldova, Ghana, Tanzania, Ethiopia, and Mozambique—have scaled up YFS, although weak health systems and inadequate financial investments often stymie sustainability.^7,10^ While the YFS in Ethiopia facilitated access to contraception in a safe environment (a YFS unit/room within health centers), contraceptive access was restricted to short-acting methods. Young people opting for the highly effective long-acting reversible contraceptives (LARCs)—implants and intrauterine devices—were referred to the main FP unit. Biddlecom et al report that in 2017, 90% of modern methods used were short-acting contraceptives while LARCs and permanent methods accounted for the remaining 10% among adolescent women in developing countries.^19^

The situation in Ethiopia, the second most populous country in sub-Saharan Africa, is reflective of this trend. The 2016 Demographic and Health Survey^20^ reports that among Ethiopian adolescents and youth using modern contraceptives, most preferred short-acting methods. Only 6.6% of those ages 20-24 and less than 2% of those ages 15-19 used LARCs. The percentage of adolescents who gave birth or were pregnant with their first child has declined since 2000 from 16% to 13%. At the same time, the median age at first birth among women ages 25-49 remained stagnant at 19.2 years, implying that half of Ethiopian women ages 25-49 gave birth for the first time before the age of 20. However, among currently married women, the total demand for FP since 2000 has risen from 44.3%^21^ to 52.5%^20^ among 15-19-year-olds and from 45.3%^21^ to 57.3%^20^ among 20-24-year-olds. Over the past 2 decades, the total fertility rate has declined—yet teenage pregnancy rates remain consistently high.

The Ethiopian Government’s commitment to improving the health and well-being of adolescents and youth is exhibited through policies, guidelines, and financial reforms that contain directives toward a multi-sectoral and integrated approach to improve quality, equity, and make YFS a priority. Ethiopia’s earlier*National Adolescent and Youth Reproductive Health Strategy: 2007–2015*^22^ and its current *National Adolescent and Youth Health Strategy (2016-2020)*^23^ recognize that YFS, which offer adolescents and youth expanded voluntary contraceptive method choice in a safe environment, is a viable approach for meeting the health needs of all young Ethiopians—and particularly their RH needs. The financial reform strategy emphasized revenue retention and increased financial autonomy of health facilities through the introduction of a financial governance system including the health facility administration board, which has the authority to raise internal revenues and generate income.^24,25^ Pursuant to Ethiopia’s Health Reform Bill,^24,25^ woredas and health facilities have therefore been empowered to generate income through personal contributions, fundraising activities, and out-of-pocket payments.

Health services in Ethiopia are primarily financed from 4 sources: the federal and regional governments; grants and loans from bilateral and multilateral donors; NGOs; and private contributions/out-of-pocket payments for services rendered. Despite significant improvement over the years, healthcare financing continues to be a major challenge in Ethiopia. The national government’s budgetary allocation of 70%-80% for essential drugs/clinical services and 20%-30% for quality improvement/preventive services inclusive of maternity care, immunizations, strengthening and promotion of adolescent and youth health, and FP including LARCs continues as the modus operandi. The Regional Health Bureau (RHB) serves as the platform for decision-making on resource allocation and planning of government and partner contributions The RHBs are charged with preventing duplication of resources and building an integrated regional implementation plan. Ethiopia has included adolescent and youth health in its normative documents,^22,23^ though without dedicated financial resources.

The LARCs and Youth Project was launched in 2014 to strengthen accessibility for an expanded contraceptive method mix for all sexually active Ethiopians under 25 years of age in a safe environment – the YFS unit. The project was a collaboration between 2 US Agency for International Development (USAID)-funded programs—the global Evidence to Action^ [1]^ Project and the bilateral/national Integrated Family Health Program Plus (IFHP+)^ [2] ^. The LARCs and Youth Project sought to offer an expanded contraceptive method mix to all sexually active women below 25 years of age at YFS units in a ‘one-stop shop’ with the other health services. The project started with a proof-of-concept stage, which was followed by a proof-of-implementation of scale-up phase. The proof of concept, or pilot experience, tested a YFS model that offered youth an expanded method choice, including LARCs, in a ‘one-stop shop’ by training YFS providers (health officer, nurse or midwife) at selected YFS units in Amhara and Tigray to counsel on and provide all contraceptive methods in one location (the YFS unit) to sexually active young persons under 25. At the same time, peer educators were trained to reach young Ethiopians with information about contraception, seeking to dispel myths and misperceptions about LARCs.^26,27^ IFHP+ in partnership with the Ethiopian Federal Ministry of Health (FMoH) and RHBs, scaled up the tested YFS delivery model^26,27^ to additional YFS units across Ethiopia (implementation of scale-up phase) from September 2015. YFS providers were trained to provide LARCs services, and peer educators were trained to dispel myths and misperceptions about LARCs. Documentation of the proof-of-implementation of scale-up phase contributed rich understanding of the experience including lessons learned that are anticipated to be used to inform national implementation and scale-up, in alignment with Ethiopia’s healthcare financing reforms,^24,25^ 2017 FP2020 commitments,^28^ and *National Adolescent and Youth Health Strategy (2016-2020).*^23^

It is important to note that the terms “spread” and “scale-up” have been used interchangeably in some implementation science literature. The literature distinguishes between the 2 terms by referring to “spread” as adoption and replication with little modification and “scale-up” as encompassing systemic/infrastructure issues. Some literature further disaggregates “scale-up” as “vertical” scale-up (institutionalization of scale-up) and “horizontal” scale-up (expansion).^29,30^ In this paper, we use the term “scale-up” to describe expansion of the tested intervention to additional YFS units without any modification/s to the tested intervention model and “implementation of scale-up” as the processes that contributed to scaling up to additional YFS units.

This paper describes successes, challenges, and barriers underpinning the proof-of-implementation of scale-up experience of the LARCs and Youth Project in scaling up the tested YFS delivery model^26,27^ to additional YFS units in Amhara and Tigray. The objectives of this paper are to assess factors that enabled or hindered scale-up, ascertain trends in LARCs uptake, and explore factors enabling sustainability.

## Methods

The study adopted a mixed methods design involving face-to-face semi-structured key informant (KI) interviews and data extraction from the Health Management Information System (HMIS) FP registers in Amhara and Tigray. The researchers conducted 56 interviews over 3 months (August-October 2017) to ascertain facilitating factors, challenges, and barriers the LARCs and Youth Project encountered when scaling up to additional YFS units and that affected sustainability. Data on contraceptive uptake at YFS units where the YFS delivery model was scaled up during the 16-month (September 2015–December 2016) proof-of-implementation of scale-up phase were extracted from the FP registers.

### Study Area and Setting

The tested YFS delivery model^26,27^ was scaled up to 182 YFS units in 4 regions: Amhara (n = 55), Tigray (n = 52), Oromia (n = 49), and Southern Nations, Nationalities and Peoples (n = 26) between September 2015 and December 2016. Systemic health systems and service delivery concerns that were external to the scaling-up model adopted included trained staff^ [3]^ turnover and absences, poor quality of care, commodity insecurity, and data quality problems. These challenges were not addressed as a component of the planning and/or execution of the scaling-up strategy. It is important to note that during the pilot test phase, commodity security, retention of YFS providers, and data quality were carefully regulated in intervention and non-intervention YFS units although only LARCs trained YFS providers and peer educators were available at the intervention YFS units.^26^

### Analytic Framework

Six elements, drawn from several sources,^1-4^ comprise the analytical framework used to document scale-up and sustainability of the ‘one-stop-shop’ YFS delivery model:

1. Stakeholder engagement
2. Roles and responsibilities
3. Policy environment
4. Financial resources
5. Quality of voluntary FP services (counseling and service provision)
6. Data availability and use

Each element constitutes an intrinsic component of planning and implementing a scale-up strategy. The 6 elements are closely linked (Table 1). Four of the 6 elements—stakeholder engagement, roles and responsibilities, policy environment, and financial resources—were used to delineate the processes involved in gaining and sustaining buy-in to scale up. Quality of voluntary FP services (counseling and service provision)^31^ and data availability and use were used to assess implementation success as a service delivery capacity function.

**Table 1:** Definitions of the Analytical Framework’s 6 Scale-Up Elements

**Scale-up Element**	**Definition**
Stakeholder engagement	The process by which organizations involve people who may be affected by the decisions made or can influence the implementation of decisions to develop a common understanding and agree on solutions that help drive long-term sustainability
Roles and responsibilities	The specific function/s and associated responsibility in performing the designated function/s
Policy environment	Accessible national policy and/or guidelines supporting the intervention being scaled up
Financial resources	Abiding interest, obligation, and responsibility for contributing funds for scale-up implementation and integrating those costs in annual budgetary expenditures
Quality of voluntary FP services	Counseling and service provision directly influencing contraceptive uptake at service-delivery outlets. For young clients, quality of care includes ensuring a separate space to maintain privacy and confidentiality and skilled service providers that offer YFS for expanded method choice at one site
Data availability and use	To assess performance and arrive at solutions for addressing poor performance by ensuring that public-sector and implementing partners provide supportive supervision to ensure quality, age-disaggregated data collection, analysis, and review at each of the primary healthcare delivery tiers

Abbreviations: FP, family planning; YFS, youth-friendly service.

### Sampling

The researchers used a multi-stage convenience purposive sampling technique to select the individual woreda-health center dyad unit – the health center aligned with its respective woreda. Amhara and Tigray regions met the purposive selection criteria of feasibility and practicality for day-to-day project oversight as these regions were directly managed by the in-country research partner, Pathfinder International, and were where the proof of concept took place.

Figure shows Amhara and Tigray primary healthcare delivery systems and within them the primary sampling frames used in this study. Administratively, there are 11 zones, 167 woredas, and 520 health centers in Amhara and 7 zones, 52 woredas, and 218 health centers in Tigray (row 1). IFHP+ operated in 6 zones, 77 woredas, and 407 health centers in Amhara and 5 zones, 35 woredas, and 141 health centers in Tigray (row 2). The ‘one-stop shop’ YFS model was operational in 55 health centers in 47 woredas and 6 zones in Amhara, and 52 health centers in 31 woredas and 5 zones in Tigray, as of December 2016 (row 3). It is important to note that this is a retrospective study of the scaling up project executed by IFHP+ and Relief Society of Tigray (REST) over a 16-month period (September 2015–December 2016). The sampling frame of 75 sites (Amhara = 36 and Tigray = 39) includes only those sites that USAID has continued to support since January 2017 under a new project^ [4] ^, and are considered accessible in terms of security risk designation, excluding any sites that were part of the proof of concept study (row 4). The zones, woredas, and health centers were randomly selected to reach the sample size of 4 aligned zones, woredas, and health centers in Amhara (IFHP+ supported) and Tigray (half IFHP+ supported and half REST supported) (row 5).

**Figure:**
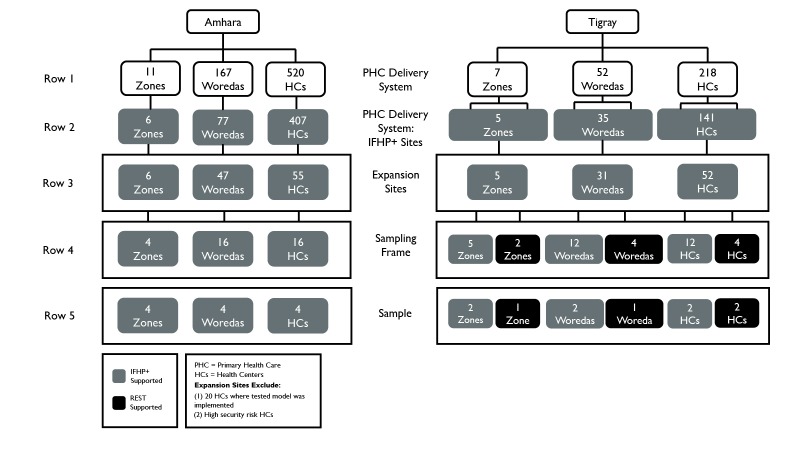
Flow Chart Illustrating the Total Number (Row 1) and Sampled (Row 5) Zones, Woredas and Health Centers in Amhara and Tigray. Abbreviations: IFHP, Integrated Family Health Program; PHC, primary healthcare; REST, Relief Society of Tigray.

### Method of Data Collection

The study involved qualitative and quantitative data collection approaches. The qualitative component entailed interviews with senior managers and technical staff from 2 sources—the public sector (RHB, zone, woreda, and health center) and implementing partners (IFHP+ and REST). The Tigray sample excluded zonal level interviews as the Tigray operational organogram excludes zonal staff. Interviews in Tigray were conducted in 3 woredas as one IFHP+ and one REST health center were situated in the same woreda.

The semi-structured interviews contained a series of questions aligned with the 6 elements of the analytical framework described earlier, including respondents’ reflections on successes/achievements, challenges, and barriers. Three semi-structured interview guides for senior managers and technical staff participants at (1) RHBs, zonal, and woreda levels; (2) health centers; and (3) implementing partners’ regional (IFHP+ and REST) and center^ [5]^ (IFHP+) levels were pre-tested, revised, and finalized during a 5-day training workshop. Six research assistants (a team leader and 2 interviewers per region) were trained on study objectives, study design, data collection instruments, the need for quality data, and principles and procedures related to human subject research. Identification and recruitment of the respondents commenced during the 5-day training workshop and continued during the data-collection period (August-October 2017). Senior managers and technical staff were identified from the sampled sites based on positions held during the scale-up phase irrespective of whether they were working in the same position as when the model was scaled up or had transferred to a different position at study recruitment.

The research team conducted 56 KI interviews—43 public-sector and 13 implementing partner interviews—interviewing 25 senior managers and 31 senior technical staff (maternal and child health [MCH] officers, youth advisors, FP advisors, and monitoring and evaluation advisors). Each interview lasted between 60-90 minutes. The researchers carried out the interviews in Amharic or Tigrigna, respectively, and used a digital recorder, for transcription and translation into English (Table 2).

**Table 2:** Number of Public Sector and Implementing Partner KI Interviews Conducted in Amhara, Tigray, and Addis Ababa; Ethiopia (August–October 2017)

**Health Administrative Levels**	**Management**	**Technical**	**Total**
Amhara			
RHB	1	1	2
Zone (n = 4)	4	4	8
Woreda (n = 4)	4	4	8
Health Center (n = 4)	4	4	8
Tigray			
RHB	1	2	3
Woreda (n = 3)	3	3	6
Health Center (n = 4)	4	4	8
Implementing partners			
Regional Office/IFHP+^a^	2	5	7
Regional Office/REST^b^	1	2	3
Center Office/IFHP+^c^	1	2	3
Total	25	31	56

Abbreviations: KI, key informant; RHB, regional health bureau; IFHP, Integrated Family Health Program; REST, Relief Society of Tigray. ^a^ IFHP+: Amhara, Tigray regional offices. ^b^ REST: Regional office; Tigray. ^c^ IFHP+: Center Office/Addis Ababa.

The quantitative component entailed data extraction from the public-sector FP registers maintained at the 8 health centers. Data extraction forms for specific FP service statistics were reviewed and subsequently pretested to assess ease of data abstraction from the scanned FP register pages. LARCs training for YFS providers occurred during different months between September 2015 and December 2016 for Amhara (n = 55) and Tigray (n = 52) YFS units. The training month for each health center was identified. The research team scanned the national FP register’s service statistics from health centers 6 months prior to the training month, the training month itself, and 6 months after the training month, for a total of 13 months of FP service statistics. Relevant data (age, acceptor status/new vs. repeat, method uptake) were extracted from these scanned pages and transferred to Excel spreadsheets to ensure standardized data extraction across all 8 health centers. The results presented in this paper are restricted to new acceptors, defined as a client who, at the current visit, accepted a FP method for the first time irrespective of discontinuation for pregnancy or any other reason.

### Data Analysis

The data analysis and results presented are descriptive, based on the 6 elements synthesized from various sources as described in our analytic framework. The research team reviewed transcripts for each administrative level (region, zone, woreda, and health center) and identified recurring themes within each of the 6 elements by triangulating data at each administrative level and across administrative levels. These recurring themes were also triangulated with the information gleaned from the implementing partners’ (IFHP+ and REST) transcripts to provide an integrated portrayal of the processes involved, successes, and challenges faced during implementation of scale-up. Finally, the KI findings were triangulated with the FP service statistics to ascertain concordance. FP register data were analyzed for 2, 6-month time periods: before and after the LARCs training month. The researchers grouped new acceptors as LARCs users and users of short-acting methods (oral pills, injectables, and condoms). Analysis of service statistics and *t* tests of association were conducted to assess subsequent shifts in uptake of LARCs and short-acting methods (6 months before and 6 months after training). Frequency distribution and binary analysis (before versus after training phase) are described. The data were analyzed using SPSS version 22.

## Results

The following section reports qualitative findings in relation to the 6 elements of our analytic framework described earlier (Table 1). This section elaborates on the perspectives of senior managers and technical staff^ [6]^ from the public sector (n = 43) and implementing partners (n = 13), juxtaposing their perspectives with quantitative findings extracted from FP registers (LARCs new acceptors before and after LARCs training).

### Stakeholder Engagement

The structure and protocols established by Ethiopia’s national health system and healthcare financing reform guidelines describe stakeholder engagement protocols. The FMoH and the RHB focus on policy, strategy, and technical support, while lower administrative levels focus on overseeing management and implementation of policy and strategy at health centers and other health facilities. In parallel, the principal implementing partner, IFHP+, oversaw and provided supportive technical assistance aligned with central and regional strategies, workplans, and budgets. This symbiotic relationship between public sector and implementing partners coalesced in the formation of regional technical working groups^ [7] ^, which were the platform for technical discussions, work plan approvals, and oversight. “*The implementing organization* […IFHP+ and REST…]* has to communicate with the RHB about their plan. It is after the agreement that the lower health administrative levels are communicated by the RHB. That is the usual flow for any program implementation* ” – Senior Manager/Woreda.

A key facilitating factor that supported stakeholder involvement was the government prioritizing^22,23^ adolescent and youth health in national strategies and guidelines “*It is part of the policy to address youth reproductive health needs* ” – Senior Manager/RHB Bureau. Other facilitating factors included evidence from pilot testing the strengthened YFS delivery model and the trusted relationship between IFHP+ and the public health system, nurtured over a decade. IFHP+ sought formal approvals by presenting the results of the pilot and the scale-up plan to the respective regional technical working groups. The formal approval letter, signed by the RHB, designated the selected zones, woredas, and health centers, the public sector implementers. “*The IFHP+ representatives discussed with the RHB and reached agreement. Then the MCH officer was informed by the RHB to discuss the plan with the implementer* […public sector staff at zone, woreda and health center…]”– Senior Technical Officer (MCH Officer)/RHB. Thereafter, IFHP+ technical staff visited the respective zones, woredas, and health centers to formally discuss implementation of the scale-up plan.

The RHB technical working groups monitored project implementation at its quarterly meetings, while the lower administrative level interacted routinely with implementing partners, health center directors, and YFS focal persons, thereby supporting government ownership, scale-up, and sustainability. Quarterly joint review meetings allowed for intensive collaboration and mutual understanding regarding achievements, barriers faced, and their resolutions. “*Review meetings were held in the presence of the representatives of these partners. The achievements and the limitations related to the implementation of activities would be discussed and ways forward would be set* ” – Senior Technical Officer (RH Officer)/REST. These review meetings aligned partner activities, prevented overlap, sustained government ownership, and resulted in strengthened collaboration among stakeholders.

Generally, a trusted working relationship existed between the public health system and implementing partners, though informants offered examples where misunderstandings occurred and were mutually resolved. For example, IFHP+ supervisory visits were to be conducted jointly with the respective woreda technical team, but the supervisory visit schedule conflicted with prior woreda team appointments. “*We had to miss some appointment with them for discussion, evaluation, and supervision because of emergency situations we have been facing and overlap of activities. We commented that they have to let us know ahead of time* ” – Senior Technical Officer (MCH Officer)/Woreda. IFHP+ staff alluded to staff turnovers and stock-outs that disrupted YFS continuation. “*Some trained professionals had to leave the service for various reasons and there were some interruptions of the service and the methods as well although briefly* ” – Senior Manager/IFHP+.

After IFHP+ phased out in December 2016, other organizations did not support LARCs training of YFS providers. However, RHB staff were confident that ownership and sustainability of the YFS should fall squarely under their mandate. “*We have owned the LARC service and we are trying to make it sustainable through training more professionals. Whether IFHP+ stays or not, the RHB considers the service* […YFS …]*as its main agenda* ” – Senior Technical Officer (MCH Officer)/RHB. On the other hand, zonal and woreda senior manager and technical staff were of the view that RHBs must invest in training service providers to ensure strengthened YFS across regions. “*The health sector has to own the LARC service for young people at all health facilities and plan for training, supply, and supervision* ” – Senior Technical Officer (MCH Officer)/Woreda. Woreda stakeholders encouraged health center directors to convince their respective health administrative boards to integrate LARCs services at YFS units. Seven health centers and a primary hospital trained the incumbent YFS providers on provision of LARCs.

### Roles and Responsibilities

The RHB, under its mandate to facilitate a favorable environment for policy and programmatic discussions, served as the primary entity responsible for overseeing the implementation of the approved scale-up plan and seeking additional FMoH funding for sustaining RH services including LARCs training, irrespective of implementing partner support. Zone and woreda staff were responsible for implementing the approved scale-up plans and conducting program monitoring activities. The zonal health department formalized the scale-up implementation process by circulating a letter to selected woredas informing them of the scale-up plans and requesting the woredas to select the health centers. Selected health centers worked with woreda staff to select potential trainees. Woreda staff assigned trained professionals to YFS units and organized necessary space for the YFS unit. Health centers were responsible for staffing their YFS units with trained staff and ensuring supportive supervision and commodity security. *“Our roles were creating supportive environment for the service including recruiting providers and assigning them at the YFS unit after training, preparing the room for the service, supervising the service, reporting the activities, and requesting for method choices* ” – Director/Health Center. Implementing partners focused on strengthening service delivery by supporting trainings and providing equipment, supplies, and supportive supervision. *“Accordingly, the roles of the IFHP+ in the LARCs implementation were training health professionals and peer educators, providing gap filling for FP commodities, furnishing the YFS centers, giving other resources such as TVs, coffee tables, periodic support to peer educators, and supervising the service”* – Senior Manager/RHB. These health center activities were planned jointly by RHB and the implementing partners.

### Policy Environment

Ethiopia’s policy environment was fully supportive of offering the full range of contraceptives, including LARCs, to adolescents and youth.^22,23^ “* There were no barriers to implementing the YFS at the policy level. The policy is supportive* ” – Senior Manager/RHB. Interviews with public-sector senior managers and technical staff and the implementing partner across all administrative and service delivery levels confirmed that national policy and guidelines support offering LARCs at YFS units and acknowledged the reproductive rights of young persons to access all contraceptive methods. “*The policy of the government is that all clients, including young people, have the right to access FP service and the method they choose based on the information given to them”* – Senior Technical Officer (MCH Officer)/RHB.

On the other hand, respondents addressed implementation challenges at administrative and service delivery levels—lack of commitment and inadequate budgetary allocations impeding scale-up and sustainability—while also indicating that the onus for bolstering commitment and committing financial resources for strengthening YFS lies with the public sector. “*Resource allocation to the YFS and RH services of the young people is limited. This may be related to budget limitation and prioritizing other health problems over youth RH service, or lack of commitment* ” – Senior Technical Officer (MCH Officer)/Woreda. Implementing partners recognized these barriers and indicated low public-sector commitment and tendency to transpose responsibility to NGOs as a hindrance to scale-up and sustainability. “*There are gaps with the implementation and strategy. For example, there should be YFS centers at all health facilities but when it comes to reality there is a budget constraint. The public sector practically gives little attention to YFS and wants it to be done by external bodies such as NGOs* ” – Senior Technical Officer (Youth RH Officer)/IFHP+ Regional.

### Financial Resources

Public-sector investments contributed the largest share of financial resources. These investments were inclusive of large capital and operating expenditures, such as construction costs, furniture, salaries, commodities, and supplies. Implementing partner contributions were limited to training costs and commodity provision to allay commodity stock-outs; *“Our support to the public sector is like a drop in the ocean. The ocean is the public sector because the health providers are there, the facility is already there* ” – Senior Manager/IFHP+ Centre Office.

Woreda staff recognized that financial responsibility rests with the public sector, which results in some budgetary constraints. “*It is the responsibility of the government to finance the service* […LARCs training…]*. However, there is the limitation of budget* ” – Senior Technical Officer (MCH Officer)/Woreda. Woreda and health facility budgets do not have separate FP or YFS budget line items; rather, they contain a more general budget line for “preventive services” (eg, MNCH [Maternal, Newborn, Child Health], FP, immunization, RH, nutrition, malaria, and tuberculosis). “*There is no budget line and budget code to allocate for specific activities like strengthening FP; … The allocation is for all prevention activities, including MNCH* ” – Senior Technical Officer (MCH Officer)/Woreda. Consequently, allocating financial resources for specific preventive services considered low priority remained challenging. “*It is unthinkable … cognizant to the critical shortage of the financial resources it would not be possible to allocate budget for specific services like FP* ” – Senior Manager/Woreda. On the other hand, budgetary allocations for capacity building and biannual review meeting workshops are included for all health-related activities. “*They* […government…] *do have a budget allocation for overall capacity building and hosting review meeting workshops on bi-annual and sometimes quarterly basis* ” – Senior Technical Officer (MCH Officer)/REST. This indicates that there is an approved funding stream applicable to Adolescent and Youth Reproductive Health and LARCs training, including trainings for peer educators.

Financial reforms and a supportive policy environment notwithstanding, little understanding of financial reforms, budgetary challenges, adherence to financial protocols, low priority and commitment were perceived as the main hinderances to scale-up and sustainability.

*“Resource allocation to the YFS and RH services of the young people is limited. This may be related to budget limitation and prioritizing other health problems over youth RH service, or lack of commitment* ” – Senior Technical Officer (MCH Officer)/Woreda.

“*There is lack of attention to youth RH problems at all levels. It is not considered as one of the most important health issues* ” – Senior Technical Officer (MCH Officer)/Woreda.

Informants from the zones, woredas, and health centers cited income-generation activities led by health centers as a mechanism for raising additional funds for health centers. This type of financing is sanctioned by the current healthcare financing law.^24,25^ However, allocation of the funds generated through health center revenues are subject to decisions made by health center administrative boards and need to comply with defined RHB financial protocols. “*Using some money from the 30% of revenue to fulfill commodities and materials for YFS needs much effort to convince the board* ” – Senior Manager/Woreda. Although the reformed healthcare financing law and national Adolescent and Youth Reproductive Health strategy should have galvanized the public sector to address young persons’ health, these have not significantly influenced health centers’ budgetary allocations. Woreda and health facility administration often remain hesitant to allocate funds for YFS, implying that there should be continued and sustained advocacy for accepting complete ownership of the “one-stop-shop” YFS approach at all administrative and service delivery levels: “*Influencing the allocation of resources for YFS is the task that needs to be the attention of the public sector* ” – Senior Manager/IFHP+ Regional Office.

### Quality of Voluntary Family Planning Services

Senior management, technical staff, and service providers mentioned 4 interlinked factors as contributors to quality of LARCs/FP services: providers (training, commitment and staff availability); access to separate space for YFS unit; sustainable commodity supplies; and supportive supervision with timely feedback.

Private and confidential YFS need to be offered in a separate room where young persons can receive preventive RH services, including LARCs. Young clients were referred to the MNCH unit if they opted for LARCs, potentially breaching confidentiality and dissuading young clients from using LARCs. Interviewees noted that young clients’ acceptability of LARCs rose when they learned that those services were offered at a separate YFS unit in a one-stop shop. “*The youth do not want to see other faces in other rooms. They prefer to get the services in one room and by one provider because it minimizes the possibility of disclosure* ” – Senior Technical Officer (MCH Officer)/Woreda.

Prior to LARCs training, the YFS providers did not provide balanced counseling inclusive of potential side-effects; rather, they restricted their counseling and service provision to short-acting methods. “*There was a gap in counseling for all FP options and the clients were influenced to choose only among the short-acting FP methods”* – Senior Technical Officer (MCH Officer)/Woreda. Respondents were cognizant that if a client was not aware of the side-effects, it might influence method continuation, resulting in removal or misperceptions.

Regional trainers conducted nationally approved, standardized, competency-based LARCs training in collaboration with IFHP+. “*We have taken good training that equipped us with the confidence and skill of counseling and inserting the methods* ” – YFS Provider/Health Center. Respondents confirmed that selection criteria included motivated, high-performing preferably female providers, committed to working at YFS units for a minimum of one year post-training. “*We tried to select better performing and motivated health professionals for the training* ” – Senior Technical Officer (MCH Officer)/RHB. Senior management and technical staff from health centers, RHBs, and implementing partners acknowledged that the ability to offer young people expanded method choice in a ‘one-stop-shop’ unit had contributed to increased LARCs uptake, reflecting improvements in quality of care. “*But after I took the training my approach and the way I deal with the youth improved* ” – YFS Provider/Health Center. However, respondents recognized that staff turnover and staff availability outside routine clinic times and on weekends continued to impede quality. “*We also have unavoidable staff turnover and interruption of the service* ” – Senior Manager/Woreda. Supportive supervision with timely feedback was important in improving FP services and ensuring commodity security: “*The supportive supervision by the district health office and the implementing partner helped us improve our counseling as we receive feedback from the supervisors* ” – YFS provider/Health Center. To improve the public sector’s capacity to continue to offer high-quality FP counseling and service provision, interviewees recommended that YFS providers receive LARCs training, stock-outs be prevented, and joint supportive supervision be conducted.

### Data Availability and Use

Health facilities record FP service statistics in national FP registers, disaggregating data by age,acceptor status (new and repeat),place of residence, and method type. Monthly data from service-delivery points are aggregated in the ‘Monthly Service Delivery Report Form,’ and submitted to the respective woredas where the data are reviewed, compiled, and submitted to the zonal health department and then to the RHB. RHBs conduct zone, woreda and health facility performance reviews quarterly, providing written and oral feedback. Respondents acknowledged that data generated from the HMIS were used for decision-making although perceptions on performance review frequency, feedback, and availability of disaggregated data varied. “*The first issue in quarter meeting* […performance review meeting…] *is whether we are achieving the […LARCs utilization…] rate or not. Our plans will then be based on this if the utilization rate is low, we have to improve and expand the services. The data is useful for planning and decision making* ” – Senior Technical (MCH Officer)/Woreda.

Challenges encountered included poor data quality (over- or under-reporting). “*There are gaps when you crosscheck the data that was reported from the facility with what is actually registered. Often, what is reported is higher than what is registered in the registration book* ” – Senior Technical (Youth Program Officer)/IFHP+ Center Office. In particular, it was reported that age- and method-specific disaggregated data are not available; although the national FP register has an age column, the compiled monthly and/or quarterly reporting format did not. “*We are analyzing the data at woreda level. Thus, we can’t identify the trends of the service use among the youth per facility. Besides, though there is age disaggregated data, the analysis of the data at regional level is not age disaggregated, which hinders analyzing the trend of the service use among the youth* ” – Senior Technical (MCH Officer)/RHB.

### Long-Acting Reversible Contraceptives Uptake

Overall, qualitative findings depicted an increase in LARCs uptake at administrative and service delivery levels. For example, the MCH Officer at the RHB alluded to the increase in LARCs uptake among young girls as a significant achievement. *“There is increasing uptake of LARC by the young girls from almost nothing* ” – Senior Technical (MCH Officer)/RHB. Service providers commented on the shift in method choice; “*Before there was the trend of highly utilizing short-acting FP methods like that of the one used every 3 months and the like but now there is an increase in the utilization of long-acting FP methods* ” – Director/Health Center. However, quantitative results comparing new LARCs acceptor uptake before and after training depicted a varied pattern: a statistically significant increase in 2 health centers, a non-significant increase in 3 health centers, and non-significant decline in 3 health centers. For example, the health center director remarked on increased awareness and utilization as demonstrated by the increase in LARCs uptake. *“The awareness and the utilization of LARCs have increased. We are now giving a better service for the young women in terms of LARCs and counseling. There is a significant improvement in the utilization of the LARCs by the young women since the opening of the service following the training”* – Director/Health Center. This perceived increase was substantiated by the quantitative finding: LARCs uptake increased from 37.1% to 50.7% (*P* value ≤ .05) (Table 3) However, this pattern was not observed in the other health centers. LARCs uptake declined from 50.8% to 41.6% (*P* value ≤.11). Service providers’ perceptions conflicted with quantitative findings. “*And if we try to see the age difference, there is an increase in the utilization of long-acting FP methods among adolescents* ” – Director/Health Center.

**Table 3:** Frequency Distribution of New Acceptors by Method Uptake (LARCs and Short-Acting Methods^a^) Disaggregated by Intervention Period^b^

**Intervention Period**	**Before** **No. (%)**	**After** **No. (%)**	***P*** **Value**
Health Center - 1			.86
LARCs	6 (10.2)	10 (11.1)	
Short-acting methods	53 (89.8)	80 (88.9)	
Health Center - 2			.66
LARCs	29 (54.7)	44 (58.7)	
Short-acting methods	24 (45.3)	31 (41.3)	
Health Center - 3			.17
LARCs	44 (28.6)	26 (21.3)	
Short-acting methods	110 (71.4)	96 (78.7)	
Health Center - 4			.05
LARCs	66 ( 37.1)	37 (50.7)	
Short-acting methods	112 (62.9)	36 (49.3)	
Health Center – 5			.11
LARCs	97 (50.8)	52 (41.6)	
Short-acting methods	94 (49.2)	73 (58.4)	
Health Center - 6			.65
LARCs	121 (46.4)	84 (48.6)	
Short-acting methods	140 (53.6)	89 (51.4)	
Health Center - 7			.52
LARCs	16 (22.5)	10 (17.9)	
Short-acting methods	55 (77.5)	46 (82.1)	
Health Center - 8			.01
LARCs	12 (15.2)	24 (33.8)	
Short-acting methods	67 (84.8)	47 (66.2)	

Abbreviation: LARCs, implants and intrauterine devices. ^a^ Short-acting methods: injectables, oral contraceptives, and condoms (male); emergency contraceptives not reported by any health center. ^b^ Intervention period: 6 months before (Before); 6 months after (After) LARCs Training.

## Discussion

The study presented in this article suggests that there are 2 principal barriers to scale-up and sustainability of the strengthened YFS delivery model: the inability to mobilize public financial resources for preventive FP services and poor quality of voluntary FP services at the planning and execution phases respectively. Ethiopia’s normative documents^22,23^ strategically elucidates adolescent and youth health policy advocating for adolescent and youth friendly health services including expanded method choice, though without obligating financial resources at the national and regional level. Despite the conducive policy environment, supportive stakeholders, and a favorable work environment for scale-up of the model—scaling up was heavily dependent on implementing partners funding LARCs training for service providers and peer educators. Furthermore, the anticipated impact of the scale-up strategy—statistically significant increases in LARCs uptake as demonstrated in the pilot project,^26^ only occurred at 2 of the 8 health centers, indicating the influence of weak health systems and poor quality of voluntary FP services.

Our findings indicate that staff shortages including transfers and absences, commodity insecurity, unsatisfactory HMIS data quality, and inadequate financing, key health systems strengthening building blocks, contributed to weak health systems, leading to underperforming services and lower LARCs uptake than anticipated.

The approved scale-up strategy was limited to LARCs training for YFS providers (insertion, removal and infection control) and peer educators (dispelling LARCs myths and misperceptions). Fidelity to the tested model was operationally constrained by staff turnovers and absences, paucity of LARCs-focused supportive supervision and data quality – components of the tested intervention.^26^ While fidelity to the tested model is optimal, the reality of scaling up in a resource-constrained country with a weak health system hindered fidelity. We acknowledge that the rigor involved in conducting and documenting the pilot phase^26^ was substantially reduced during scaling up and might have contributed to our results. Notwithstanding contributing influencers and barriers to scaling up a tested intervention model, scaling-up efforts must always be accompanied with research documenting planning and implementation processes, health-related outcomes, and impact to communicate lessons learned and opportunities for strengthening scale-up.^32,34^

Study findings are largely consistent with existing literature,^5,11,13-15^ indicating that mobilizing financial resources and quality of care are of paramount importance for effective scale-up and sustainability of interventions that aim to improve RH outcomes among adolescents and youth nationally. In other words, support for adolescent and youth RH was on paper, but there was limited financial allocation. Most of the preventive health financial allocations were reserved for general preventive programs rather than specific activities such as FP or youth RH programs. In fact, only 9% of Ethiopia’s total health spending went to RH services (both maternal and FP services), while 49% of spending was reserved for prevention, management, and treatment of infectious and parasitic diseases and aligned with Ethiopia’s disease burden.^35^

Recognizing the role that several factors, such as political leadership and commitment, good governance, stakeholder engagement, effective program management, technical support from NGOs, relevance, and simplicity of implementation, played in the potential for scale-up,^4-15^ the crucial driving force behind scaling up the YFS delivery model was, we suggest, the quarterly public sector technical review meetings. This collaborative platform strengthened relationships among stakeholders, aligned implementing partner activities, avoided overlap, and sustained government ownership. The supportive policy environment bolstered actions taken at higher administrative levels. National commitment was stymied at lower levels by low commitment, competition with other high-profile/priority activities, and inadequate emphasis on YFS during supportive supervision visits.

It is important to note that while the intervention being scaled up was simple and technically sound with consensus about its value, actively engaged a broad range of stakeholders, and used a phased scaling-up approach, the lingering effects of systemic bottlenecks hindered effective large-scale implementation and sustainability. While the study findings corroborate Yamey’s success factors,^4^ results also showcase that each of Yamey’s proposed success factors, while necessary, are not sufficient for effective large-scale implementation. The influence of contextual parameters, such as socio-cultural norms and beliefs, fiscal environment, quality of care, data quality and utilization, and politics of commitment, must be simultaneously addressed. Mobilizing financial resources and specifically reliance on external funding sources, need explicit attention. While in the near to medium term, reliance on external funding sources will be necessary, the need for sustainable domestic funding must be addressed. The sixth round of the Government of Ethiopia’s Health Accounts, 2013-2014,^35^ noted a rise in domestic health financing (64%) and a decline in external funding (36%). The Health Accounts, however, also recommended that Ethiopia continue increasing its domestic financing to make healthcare financing more sustainable. Government priorities and domestic financing are currently harmonized with Ethiopia’s disease burden,^35^ whereas external donor-funded interventions are earmarked for preventive services. Our results highlight this impasse, noting that despite a supportive youth-friendly policy environment, resource allocation for YFS and other prevention services continue to be externally funded. Consequently, due recognition must be given to cost efficiencies of preventive services, and the current 70/30 curative/preventive public-sector budgetary split in healthcare financing must be revisited, particularly at the lower level of the healthcare delivery system, to facilitate a smooth transition from donor-supported interventions.^15^

The study is strengthened by its use of both quantitative and qualitative methods, particularly the inclusion of method-specific contraceptive uptake before and after LARCs training. Achieving successful scale-up and sustainability depends on the independent contribution of each of the 6 elements in our analytic framework. However, discerning the actual contribution of each of these elements is difficult as these are intrinsically intertwined. For example, without a supportive policy environment, it will be difficult to invest in strengthened YFS, secure financial resources for YFS, and engage stakeholders for their support during implementation. On the other hand, even with a supportive policy environment, stakeholder engagement will not result in successful scale up of YFS if stakeholders have their own agendas and lack financial autonomy and common understanding of the importance of scaling up the ‘one-stop shop’ YFS model. Our findings illustrate that perceptions of senior managers and technical staff from the public sector and implementing partners, conflicted with the quantitative evidence. The widespread perceptions of increased LARCs uptake lends a certain degree of mistaken credibility to the potential feasibility of scaling up the ‘one-stop shop’ approach to all YFS outlets in Ethiopia. On the other hand, senior managers and technical staff views are based perhaps on their understanding distilled from the 107 health centers that have scaled-up the “one-stop shop” approach, rather than the sampled 8 health centers. Furthermore, our multi-stage convenience purposive sampling technique and selection criteria might also have inadvertently contributed to the disconnect between the interview findings and LARCs uptake. Three of the 6 health centers with non-significant findings in LARCs uptake had reasonably high LARCs uptake (over 45%) before the intervention, possibly reflecting a ceiling effect – LARCs uptake among new acceptors had reached a pre-determined level prior to the intervention. Health centers and service providers were selected by the respective RHBs, zones, and woredas, without perhaps careful consideration of LARCs uptake at near threshold level as an exclusion criterion during planning and execution of scale-up to additional YFS units.

Limitations in the study design should be noted when interpreting the results.^36,37^ First, the sample was not representative due to its qualitative design. Rather, the value of the qualitative research was the rich, context-specific data generated. Another limitation of qualitative research is that data quality is heavily dependent on the individual skills of the interviewer, is more easily influenced by interviewers and interviewees personal biases including social desirability bias, and rigor is more difficult to maintain than when conducting quantitative research. For example, analysis of the data involved transcribing recorded interviews into Amharic and Tigrigna, and then translating the transcripts into English. Consequently, some of the richer contextual data may have been lost in the process of transcription and translation. Third, much of the data collected were retrospective in nature concerning training, meetings, decisions made, challenges faced, and events that happened. Consequently, information from respondents related to events which took place over the past 2 years may be incomplete, altered, or not well-recalled. Finally, the study was not designed to draw inferences or generalize about the process and outcomes of the scale-up approach in these 2 regions. Rather, it was an exploratory, descriptive study to determine what worked and what did not work, and barriers and challenges addressed in the development and implementation of the scale-up approach. Notwithstanding the lack of generalizability, the study findings provide insight into challenges faced and successes encountered when scaling up the YFS delivery model to additional health centers in the same regions where the model had been tested, its sustainability, and potential for further expansion in these regions, other regions in Ethiopia, and other countries. In essence, our study findings contribute to the significance of fidelity to the tested model with specific attention given to the contribution of weak health systems ie, staffing, supportive supervision, commodity security and data quality that were addressed in the pilot study.^26^ In addition, the study findings add to the growing body of evidence on enabling factors and barriers at the planning and execution phases of scaling up a simple intervention to increase coverage and achieve RH outcomes. By organizing elements in an analytical framework, key overall conclusions are drawn that cast light on what it takes to expand coverage and assess impact in Ethiopia and beyond.

## Conclusion

The Government of Ethiopia is fully committed to improving adolescent and youth health including FP by offering young people full contraceptive choice. Despite this national impetus, limited financial resources and health systems constraints hamper progress. Without an obligated budget line item for YFS included in regional, woreda, and health center budgets, challenges to sustaining and scaling up the tested YFS delivery model in Amhara, Tigray, and beyond will continue. Health systems need to be strengthened so that YFS units can offer adolescents and youth full contraceptive choice in a confidential, comfortable space where they can receive counseling and services by a youth-friendly trained provider that ensure voluntary informed choice. This can only be achieved by addressing human resource shortages, quality of care, commodity security, and quality data availability and use. Simply planning for a scale-up strategy without fully addressing weak health systems and availability of financial resources significantly undermines the potential of scale-up and sustainability after donor-supported project funds have ended. Excluding these elements as strategic components of a systematic scale-up approach is tantamount to being blind to the ‘*elephant in the room.* ’ While progress has been made in Ethiopia, it will still take considerable work to sustain and scale up well-functioning YFS units offering expanded method choice in a ‘one-stop shop’ model. The scale-up community must prioritize relevant health systems strengthening building blocks and financial resources as crucial elements in any systematic scale-up framework to improve RH outcomes, reduce unintended pregnancy, improve maternal health, and achieve Sustainable Development Goal 3.

## Acknowledgements

The authors thank Venkatraman Chandra-Mouli, Patricia MacDonald, Mengistu Asnake and Murtala Mai for their valuable feedback on the article. We gratefully acknowledge the work and dedication of our research teams in Amhara and Tigray: Dr. Telake Azale, Mr. Abebaw Addis, and Mr. Getu Dasash of Gondar University, Amhara; and Dr. Araya Abrha Medhanyie, Mr. Mussie Alemayehu, and Mr. Zinabu Hadis of Mekelle University, Tigray. We are very grateful for Dr. Kidest Lulu’s support during the conduct of the study. This work was supported by the USAID under the terms of Award No. AID-OAA-A-11-00024 (Evidence to Action and Integrated Family Health Program Plus).

## Ethical issues

The PATH Research Ethics Committee and the Local Regional Research Committees (Amhara and Tigray) approved the study. The research assistants involved in data collection, management, or analysis were trained to protect the privacy of participants and clients records and the need to keep all data confidential. KI interviews and data extraction from FP registers were conducted only after all participants and Director/Health Centers respectively had given informed consent.

## Competing interests

The opinions expressed in this publication are those of the authors and do not necessarily reflect the views of Pathfinder International, the United States Agency for International Development or the United States Government.

## Authors’ contributions

FFF conceived the idea and lead the write-up of the manuscript. HZ contributed to the study concept, data collection tools, data collection, the write-up and review of the manuscript. All authors read and approved the final manuscript.

## Authors’ affiliations

^1^Evidence to Action/PATH, Washington, DC, USA. ^2^Pathfinder International, Addis Ababa, Ethiopia.

## Endnotes


[1] The E2A project addresses the RH care needs of girls, women, and underserved communities around the world by increasing support, building evidence, and leading the scale-up of evidence-based practices that improve FP services.



[2] The IFHP+ project promotes an integrated model for strengthening MCH, FP, and RH services for rural and hard-to-reach populations in 4 regions of Ethiopia (Oromiya, Tigray, Southern Nations, Nationalities and Peoples and Amhara).



[3] LARCs-YF trained service providers.



[4] Transform: Primary Health Care Project, the USAID bilateral/national project implemented by Pathfinder International and John Snow Inc., commenced operations as of January 2017.



[5] REST does not operate a headquarters/center office in Addis Ababa.



[6] MCH officers, youth advisors, FP advisors, and monitoring and evaluation advisors.



[7] Members comprise technical staff from RHB; and international and national NGOs operating in the region, chaired by the Head/Deputy Head/RHB.


## Key messages

**Implications for policy makers**

- Youth-friendly services (YFSs) offering a full range of contraceptives including long-acting reversible contraceptive (LARC) methods for young people ages 15 to 24 in a “one-stop shop” approach is a feasible strategy for improving method mix in Ethiopia.
- For this strengthened family planning (FP) service delivery approach to be scalable and sustainable, health systems need to be strengthened.
- Merely planning for a scale-up strategy without fully addressing weak health systems, specifically human resource shortages, quality of care, quality data availability, commodity security, and availability of financial resources, undermines the potential for scale-up and sustainability after donor-supported project funds have ended.

**Implications for the public**

Our study results portray that although scale-up of a tested service delivery model that provides family planning (FP) counseling and services for all available contraceptive methods including long-acting reversible methods in a “one-stop shop” was well-planned and executed, the scale-up process was fraught with implementation challenges. The Government of Ethiopia has promulgated a range of supportive normative documents for adolescent and youth reproductive health (RH). Despite careful deliberation and diligent preparation for scale-up and execution of a tested youthfriendly FP service delivery model, there were challenges in rolling out the model and improving its potential for sustainability. These challenges related to staff shortages, a safe environment ensuring confidentiality of young clients, dedicated preventive financial resources and data quality. Our findings indicate that not addressing these challenges is likely to negatively impact the uptake of long-acting contraceptive methods among adolescents and youth and derail sustainability particularly when project funds ends.
